# The Tomato Sequencing Project, the First Cornerstone of the International Solanaceae Project (SOL)

**DOI:** 10.1002/cfg.468

**Published:** 2005-04

**Authors:** Lukas A. Mueller, Steven D. Tanksley, Jim J. Giovannoni, Joyce van Eck, Stephen Stack, Doil Choi, Byung Dong Kim, Mingsheng Chen, Zhukuan Cheng, Chuanyou Li, Hongqing Ling, Yongbiao Xue, Graham Seymour, Gerard Bishop, Glenn Bryan, Rameshwar Sharma, Jiten Khurana, Akhilesh Tyagi, Debasis Chattopadhyay, Nagendra K. Singh, Willem Stiekema, P. Lindhout, Taco Jesse, Rene Klein Lankhorst, Mondher Bouzayen, Daisuke Shibata, Satoshi Tabata, Antonio Granell, Miguel A. Botella, Giovanni Giuliano, Luigi Frusciante, Mathilde Causse, Dani Zamir

**Affiliations:** 1 Department of Plant Breeding Cornell University Ithaca NY USA; 2 Boyce Thompson Institute Ithaca NY USA; 3 Department of Biology Colorado University Fort Collins CO USA; 4 KRIBB Taejon Korea; 5 Seoul National University Seoul Korea; 6 Institute of Genetics and Developmental Biology Chinese Academy of Sciences Beijing 100101 China; 7 Warwick HRI Wellesbourne UK; 8 Imperial College London UK; 9 SCRI Invergowrie Dundee UK; 10 University of Hyderabad Hyderabad India; 11 University of Delhi South Campus New Delhi India; 12 National Centre For Plant Genome Research New Delhi India; 13 NRC on Plant Biotechnology Indian Agricultural Research Institute New Delhi India; 14 Centre for BioSystems Genomics Wageningen The Netherlands; 15 University of Wageningen Wageningen The Netherlands; 16 Keygene Wageningen NV. The Netherlands; 17 Plant Research International Wageningen The Netherlands; 18 Institut National Polytechnique de Toulouse Toulouse France; 19 Kazusa Chiba Kisarazu Japan; 20 Instituto de Biologia Molecular y Celular de Plantas Valencia Spain; 21 University of Malaga Malaga Spain; 22 ENEA Rome Italy; 23 U Naples Naples Italy; 24 INRA Avignon France; 25 Hebrew University Jerusalem Israel

## Abstract

The genome of tomato (*Solanum lycopersicum*) is being sequenced by an international consortium of 10 countries (Korea, China, the United Kingdom, India, The Netherlands, France, Japan, Spain, Italy and the United States) as part of a larger initiative called the ‘International Solanaceae Genome Project (SOL): Systems Approach to Diversity and Adaptation’. The goal of this grassroots initiative, launched in November 2003, is to establish a network of information, resources and scientists to ultimately tackle two of the most significant questions in plant biology and agriculture: (1) How can a common set of genes/proteins give rise to a wide range of morphologically and ecologically distinct organisms that occupy our planet? (2) How can a deeper understanding of the genetic basis of plant diversity be harnessed to better meet the needs of society in an environmentally friendly and sustainable manner? The Solanaceae and closely related species such as coffee, which are included in the scope of the SOL project, are ideally suited to address both of these questions. The first step of the SOL project is to use an ordered BAC approach to generate a high quality sequence for the euchromatic portions of the tomato as a reference for the Solanaceae. Due to the high level of macro and micro-synteny in the Solanaceae the BAC-by-BAC tomato sequence will form the framework for shotgun sequencing of other species. The starting point for sequencing the genome is BACs anchored to the genetic map by overgo hybridization and AFLP technology. The overgos are derived from approximately 1500 markers from the tomato high density F2-2000 genetic map (http://sgn.cornell.edu/). These seed BACs will be used as anchors from which to radiate the tiling path using BAC end sequence data. Annotation will be performed according to SOL project guidelines. All the information generated under the SOL umbrella will be made available in a comprehensive website. The information will be interlinked with the ultimate goal that the comparative biology of the Solanaceae—and beyond—achieves a context that will facilitate a systems biology approach.


**Comparative and Functional Genomics**
*Comp Funct Genom* 2005; **6**: 153–158.
Published online in Wiley InterScience (www.interscience.wiley.com). **DOI:** 10.1002/cfg.468

**Conference Paper**

### The Tomato Sequencing Project, the first cornerstone of the International Solanaceae Project (SOL)

†Lukas A. Mueller[1]*, Steven D. Tanksley[1], Jim J. Giovannoni[2], Joyce van Eck[2], Stephen Stack[3], Doil Choi[4], Byung Dong Kim[5], Mingsheng Chen[6], Zhukuan Cheng[6], Chuanyou Li[6], Hongqing Ling[6], Yongbiao Xue[6], Graham Seymour[7], Gerard Bishop[8], Glenn Bryan[9], Rameshwar Sharma[10], Jiten Khurana[11], Akhilesh Tyagi[11], Debasis Chattopadhyay[12], Nagendra K. Singh[13], Willem Stiekema[14], P. Lindhout[15], Taco Jesse[16], Rene Klein Lankhorst[17], Mondher Bouzayen[18], Daisuke Shibata[19], Satoshi Tabata[19], Antonio Granell[20], Miguel A. Botella[21], Giovanni Giuliano[22], Luigi Frusciante[23], Mathilde Causse[24] and Dani Zamir[25]

†*A full list of affiliations appears at the end of this paper*

**Correspondence to:*
*Lukas A. Mueller, Department of Plant Breeding, Cornell University, Ithaca, NY, USA.*
*E-mail: Lam87@cornell.edu*

### Abstract

**The genome of tomato (*Solanum lycopersicum*) is being sequenced by an international consortium of 10 countries (Korea, China, the United Kingdom, India, The Netherlands, France, Japan, Spain, Italy and the United States) as part of a larger initiative called the 'International Solanaceae Genome Project (SOL): Systems Approach to Diversity and Adaptation'. The goal of this grassroots initiative, launched in November 2003, is to establish a network of information, resources and scientists to ultimately tackle two of the most significant questions in plant biology and agriculture: (1) How can a common set of genes/proteins give rise to a wide range of morphologically and ecologically distinct organisms that occupy our planet? (2) How can a deeper understanding of the genetic basis of plant diversity be harnessed to better meet the needs of society in an environmentally friendly and sustainable manner? The Solanaceae and closely related species such as coffee, which are included in the scope of the SOL project, are ideally suited to address both of these questions. The first step of the SOL project is to use an ordered BAC approach to generate a high quality sequence for the euchromatic portions of the tomato as a reference for the Solanaceae. Due to the high level of macro and micro-synteny in the Solanaceae the BAC-by-BAC tomato sequence will form the framework for shotgun sequencing of other species. The starting point for sequencing the genome is BACs anchored to the genetic map by overgo hybridization and AFLP technology. The overgos are derived from approximately 1500 markers from the tomato high density F2-2000 genetic map (http://sgn.cornell.edu/). These seed BACs will be used as anchors from which to radiate the tiling path using BAC end sequence data. Annotation will be performed according to SOL project guidelines. All the information generated under the SOL umbrella will be made available in a comprehensive website. The information will be interlinked with the ultimate goal that the comparative biology of the Solanaceae — and beyond — achieves a context that will facilitate a systems biology approach. Copyright © 2005 John Wiley & Sons, Ltd.**

Received: 31 January 2005
Accepted: 2 February 2005

**Keywords:** Tomato; *Solanum lycopersicum*; Solanaceae; SOL project

Copyright © 2005 John Wiley & Sons, Ltd.

### Introduction

The Solanaceae, also called nightshades, are a large family of more than 3000 species, including the tuber-bearing potato, a number of fruit-bearing vegetables (tomato, eggplant, peppers), ornamental plants (petunias, *Nicotiana*), plants with edible leaves (*Solanum aethiopicum, S. macrocarpon*) and medicinal plants (e.g. *Datura, Capsicum*) [1]. The Solanaceae are the third most economically important plant taxon, and the most valuable in terms of vegetable crops. They are also the most variable of crop families in terms of agricultural utility. The closely related coffee (Rubiaceae) is a highly valuable commodity worldwide. In addition to their role as important food sources, many solanaceous species play a role as model plants, such as tomato and pepper for the study of fruit development [2–9], potato for tuber development [10,11], petunia for the analysis of flavonoids, and tomato and tobacco for plant defence [12–16]. The nightshades have also attracted interest because they produce a number of secondary metabolites, some of which have medicinal properties. The Solanaceae are remarkable in that the gene content of the different species is similar despite the markedly different phenotypic outcomes, making the Solanaceae an excellent model for the study of adaptation to natural and agricultural environments [17]. Many Solanaceae share a basic set of 12 chromosomes and are diploid, indicating an absence of large genome duplications and polyploidizations during the evolutionary history of this family. These intrinsic features of the Solanaceae and closely related species such as coffee make them well suited to address fundamental questions in plant biology.

In November 2003, the International Solanaceae Project (SOL) was initiated at a meeting near Washington, where goals were established for Solanaceae research for the next decade (**http://sgn.cornell.edu/solanaceae-project/**). The ultimate aim of the SOL project is to address two of the most significant questions in plant biology and agriculture: (a) how can a common set of genes/proteins give rise to a wide range of morphologically and ecologically distinct organisms that occupy our planet?; and (b) how can a deeper understanding of the genetic basis of plant diversity be harnessed to better meet the needs of society in an environmentally friendly and sustainable manner? To answer these questions in the context of the Solanaceae, it is necessary to link traits to sequence, requiring both extensive phenotyping [18] and sequence information. It would be desirable to obtain as many full solanaceous genome sequences as possible for direct comparison. Due to the high cost of sequencing complete genomes at high quality, this is not feasible at present. The alternative is to fully sequence a high quality reference genome, and to map onto it 'cheaper' sequence data, such as ESTs, methyl-filtered sequences [19–21] or low Cot sequences [22,23] from other species. The availability of good comparative maps [24–26] between many solanaceous plants and the large numbers of EST sequences already available [27] is a great benefit in this approach. Thus, sequencing the gene-rich regions of the tomato genome will be the first cornerstone of the SOL project. After sequencing two rosids, *Arabidopsis* [28] and *Medicago* [29], sequencing a solanaceous plant will shed light on a genome of the more distantly related asterid clade, which will permit comparisons between genomes at longer evolutionary distances and thereby help define a larger view of plant evolution. All the information generated under the SOL umbrella will be made available in a comprehensive website, where all information will be interlinked such that, ultimately, the comparative biology of the Solanaceae will become available in a context that will facilitate a systems biology approach to understanding genome evolution, plant development and plant responses to the environment.

### Tomato genome structure and sequencing method

Approximately three-quarters (730 Mb) of the 950 Mb tomato genome exists as pericentromeric heterochromatin. The remaining one-quarter (220 Mb) of the tomato genome consists of the distal, euchromatic segments of the chromosomes. The DNA found in heterochromatin is rich in repetitive sequences and poor in genes, making it difficult to sequence. The euchromatin is thought to contain mostly single copy sequences and includes more than 90% of the genes, making it relatively easy to sequence. Therefore, the strategy is to sequence only the euchromatic portion of the genome to cover most of the gene space. Sequencing 220 Mb of the tomato genome is therefore a little less

Copyright © 2005 John Wiley & Sons, Ltd. 

than twice the effort of sequencing the *Arabidopsis* genome at 150 Mb [30].

The ordered BAC approach was chosen for sequencing the tomato genome. All sequencing will be done using standard BAC libraries: the well-characterized *Hin*dIII library [31], plus two additional libraries that the US part of the sequencing project will provide; all libraries are or will be deep BAC end-sequenced by the US project, for a total of 400 000 BAC end sequences. All sequence will be derived from *Solanum lycopersicum* var Heinz 1706, as this was the basis for the original *Hin*dIII library. Sequencing will be based on the F2-2000 map (**http://sgn.cornell.edu/**), which has been used as the basis for anchoring 1500 markers by overgo (overlapping oligo) hybridization [32]. Currently, more than 650 unambiguous anchor points are available, but further analysis will increase this number to an estimated 800–1000 anchor points. In addition, the F2-2000 map is being combined with an AFLP map, containing more than 1200 markers, generated at Keygene in The Netherlands, providing even more anchor points. For each anchor point, one of the many anchored BACs will be selected as a 'seed' and sequenced. The tiling path will then be generated by walking out from this seed in both directions, using the deep BAC end-sequencing data available for the BAC libraries. Information from the fingerprint contig (FPC) map available for the *Hin*dIII library will also be used. FISH analysis will be used to confirm chromosome mappings and delineate the euchromatin/heterochromatin boundaries [33]; anchored BACs will also be mapped on IL lines to verify location. Other methods available for full genome sequencing have also been assessed. Full genome shotgun sequencing is not a cost-effective way to sequence a fraction of the genome. Methyl and Cot filtering are both methods providing a bias for coding sequence and therefore euchromatic sequence. All these methods do not by themselves provide gene order, which will be critical for a reference genome.

Currently, 10 countries are involved in sequencing the tomato genome. The 12 chromosomes have been split up between the countries as follows (see Figure 1): Korea (chromosome 2), China (3), UK (4), India (5), The Netherlands (6), France (7), Japan (8), Spain (9), Italy (12) and USA (1, 10, 11). The US project will also generate the additional BAC libraries, perform BAC end-sequencing, and build a data repository as a resource for the entire project. Argentina will sequence the mitochrondrial genome, and Italy the chloroplast genome. The organellar genome sequences will be important for distinguishing genomic insertions of organellar sequences from organellar contamination contained in the BAC libraries.

### Bioinformatics

In such a large project, bioinformatics plays a crucial role. In particular, the sequence quality standards and the annotation have to be uniform across the different chromosomes generated through different national projects, and efficient data access has to be provided to the scientific community. A SOL bioinformatics committee comprised of participating sequencing country representatives is working on a bioinformatics guideline document that should be followed by all project members to ensure that results are comparable in the entire genome. The guidelines are available from **http://sgn.cornell.edu/solanaceae-project/**. In addition, the following important conventions have been adopted: (a) the original data has to be stored for all data types, such as all chromatograms and assembly files from the BAC sequencing, all supporting data for gene calls etc; (b) all data has to be traceable to the submitters and data generators; and (c) a unified web resource will be created that makes all data accessible to the users without delay and in an intuitive format ('one-stop-shop'). Several centres will work together to implement the portal, including MIPS (**http://mips.gsf.de/**), VIB Ghent, Wageningen University and the Research Centre, KRIBB (**http://www.kribb.re.kr**), Kazusa (**http://www.kazusa.or.jp/**), SGN and centres in other countries. All sequence data will be submitted to GenBank, and to the Solanaceae Genomics Network (SGN; **http://sgn.cornell.edu/**), which will serve as a repository and access point for the data.

### Conclusions

Sequencing the tomato genome offers exciting new perspectives and opportunities for plant biology. The sequence will be compared to other

Copyright © 2005 John Wiley & Sons, Ltd.

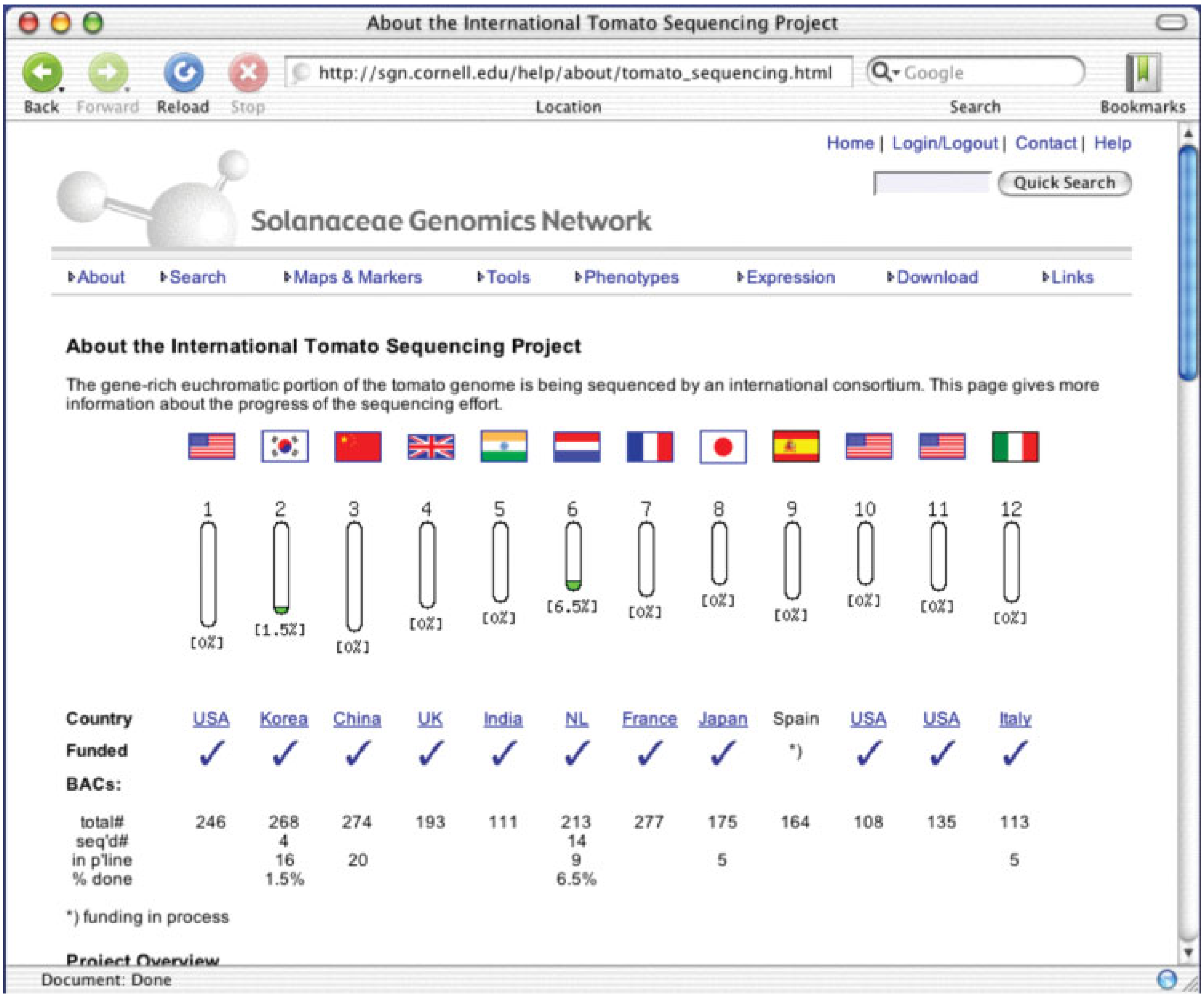

**Figure 1.** Overview of the International Tomato Sequencing Project. The 12 tomato chromosomes are sequenced by an international consortium of 10 countries [Korea (chromosome 2), China (3), UK (4), India (5), The Netherlands (6), France (7), Japan (8), Spain (9), Italy (12) and USA (1, 10, 11)], as shown. This overview is available from the SGN website (**http://sgn.cornell.edu/help/about/tomato_sequencing.html**) and will be continuously updated as sequencing progresses. More information on the project is available on the page sequenced genomes such as *Arabidopsis* and rice to inform us of the evolutionary history of these plants. In conjunction with sequence data for other solanaceous plants, the tomato sequence will be the basis for investigating the phenotypic diversity and comparative biology in the Solanaceae, one of the main aims of the SOL project. This will shed light on mechanisms of gene regulation, evolution, signalling, disease resistance and defence, and fruit development and quality, and finally contribute to the improvement of agriculture through the phenotypic diversity found in nature.

### Full List of Author Affiliations

1. Department of Plant Breeding, Cornell University, Ithaca, NY, USA
2. Boyce Thompson Institute, Ithaca, NY, USA
3. Department of Biology, Colorado University, Fort Collins, CO, USA
4. KRIBB, Taejon, Korea
5. Seoul National University, Seoul, Korea
6. Institute of Genetics and Developmental Biology, Chinese Academy of Sciences, Beijing 100101, China
7. Warwick HRI, Wellesbourne, UK

Copyright © 2005 John Wiley & Sons, Ltd. 

8. Imperial College, London, UK
9. SCRI Invergowrie, Dundee, UK
10. University of Hyderabad, Hyderabad, India
11. University of Delhi South Campus, New Delhi, India
12. National Centre For Plant Genome Research, New Delhi, India
13. NRC on Plant Biotechnology, Indian Agricultural Research Institute, New Delhi, India
14. Centre for BioSystems Genomics, Wageningen, The Netherlands
15. University of Wageningen, Wageningen, The Netherlands
16. Keygene, NV., Wageningen, The Netherlands
17. Plant Research International, Wageningen, The Netherlands
18. Institut National Polytechnique de Toulouse, Toulouse, France
19. Kazusa, Kisarazu, Chiba, Japan
20. Instituto de Biologia Molecular y Celular de Plantas Valencia, Spain
21. University of Malaga, Malaga, Spain
22. ENEA, Rome, Italy
23. U Naples, Naples, Italy
24. INRA, Avignon, France
25. Hebrew University, Jerusalem, Israel

### References

1. Knapp S. 2002. Tobacco to tomatoes: a phylogenetic perspective on fruit diversity in the Solanaceae. *J Exp Bot.* **53**(377): 2001–2022.
2. Tanksley SD. 2004. The genetic, developmental, and molecular bases of fruit size and shape variation in tomato. *Plant Cell.* **16**(suppl): S181–189.
3. Alexander L, Grierson D. 2002. Ethylene biosynthesis and action in tomato: a model for climacteric fruit ripening. *J Exp Bot.* **53**(377): 2039–2055.
4. Giovannoni JJ. 2004. Genetic regulation of fruit development and ripening. *Plant Cell.* **16**(suppl): S170–180.
5. Adams-Phillips L, Barry C, Giovannoni J. 2004. Signal transduction systems regulating fruit ripening. *Trends Plant Sci.* **9**(7): 331–338.
6. Brummell DA, Harpster MH. 2001. Cell wall metabolism in fruit softening and quality and its manipulation in transgenic plants. *Plant Mol Biol* **47**(1–2): 311–340.
7. Hamilton AJ, Fray RG, Grierson D. 1995. Sense and antisense inactivation of fruit ripening genes in tomato. *Curr Top Microbiol Immunol* **197**: 77–89.
8. Gray J, Picton S, Shabbeer J, Schuch W, Grierson D. 1992. Molecular biology of fruit ripening and its manipulation with antisense genes. *Plant Mol Biol* **19**(1): 69–87.
9. Fray RG, Grierson D. 1993. Molecular genetics of tomato fruit ripening. *Trends Genet* **9**(12): 438–443.
10. Fernie AR, Willmitzer L. 2001. Molecular and biochemical triggers of potato tuber development. *Plant Physiol* **127**(4): 1459–1465.
11. Prat S, Frommer W, Hofgen R, *et al.* 1990. Gene expression during tuber development in potato plants. *FEBS Lett* **268**(2): 334–338.
12. Pedley KF, Martin GB. 2003. Molecular basis of Pto-mediated resistance to bacterial speck disease in tomato. *Annu Rev Phytopathol* **41**: 215–243.
13. Li L, Li C, Howe GA. 2001. Genetic analysis of wound signaling in tomato. Evidence for a dual role of jasmonic acid in defense and female fertility. *Plant Physiol* **127**(4): 1414–1417.
14. Gebhardt C, Valkonen JP. 2001. Organization of genes controlling disease resistance in the potato genome. *Annu Rev Phytopathol* **39**: 79–102.
15. Bogdanove AJ, Martin GB. 2000. AvrPto-dependent Pto-interacting proteins and AvrPto-interacting proteins in tomato. *Proc Natl Acad Sci USA* **97**(16): 8836–8840.
16. Hui D, Iqbal J, Lehmann K, Gase K, Saluz HP, Baldwin IT. 2003. Molecular interactions between the specialist herbivore *Manduca sexta* (Lepidoptera, Sphingidae) and its natural host *Nicotiana attenuata*: V. microarray analysis and further characterization of large-scale changes in herbivore-induced mRNAs. *Plant Physiol.* **131**(4): 1877–1893.
17. Knapp S, Bohs L, Nee M, Spooner DM. 2004. Solanaceae — a model for linking genomics with biodiversity. *Comp Funct Genom* **5**: 285–291.
18. Menda N, Semel Y, Peled D, Eshed Y, Zamir D. 2004. *In silico* screening of a saturated mutation library of tomato. *Plant J* **38**(5): 861–872.
19. Whitelaw CA, Barbazuk WB, Pertea G, *et al.* 2003. Enrichment of gene-coding sequences in maize by genome filtration. *Science* **302**(5653): 2118–2120.
20. Palmer LE, Rabinowicz PD, O'Shaughnessy AL, *et al.* 2003. Maize genome sequencing by methylation filtration. *Science* **302**(5653): 2115–2117.
21. Fu Y, Hsia AP, Guo L, Schnable PS. 2004. Types and frequencies of sequencing errors in methyl-filtered and high Cot maize genome survey sequences. *Plant Physiol* **135**(4): 2040–2045.
22. Peterson DG, Schulze SR, Sciara EB. 2002. Integration of Cot analysis, DNA cloning, and high-throughput sequencing facilitates genome characterization and gene discovery. *Genome Res* **12**(5): 795–807.
23. Yuan Y, SanMiguel PJ, Bennetzen JL. 2003. High-Cot sequence analysis of the maize genome. *Plant J* **34**(2): 249–255.
24. Tanksley SD, Ganal MW, Prince JP, *et al.* 1992. High density molecular linkage maps of the tomato and potato genomes. *Genetics* **132**(4): 1141–1160.
25. Doganlar S, Frary A, Daunay MC, Lester RN, Tanksley SD. 2002. A comparative genetic linkage map of eggplant (*Solanum melongena*) and its implications for genome evolution in the Solanaceae. *Genetics* **161**(4): 1697–1711.
26. Fulton TM, Van der Hoeven R, Eanetta NT, Tanksley SD. 2002. Identification, analysis, and utilization of conserved ortholog set markers for comparative genomics in higher plants. *Plant Cell* **14**(7): 1457–1467.
27. Van der Hoeven R, Ronning C, Giovannoni J, Martin G, Tanksley S. 2002. Deductions about the number, organization

Copyright © 2005 John Wiley & Sons, Ltd. 

and evolution of genes in the tomato genome based on analysis of a large expressed sequence tag collection and selective genomic sequencing. *Plant Cell* **14**: 1441–1456.

28. AGI. 2000. Analysis of the genome sequence of the flowering plant *Arabidopsis thaliana*. *Nature* **408**(6814): 796–815.
29. May GD, Dixon RA. 2004. *Medicago truncatula*. *Curr Biol* **14**(5): R180–181.
30. Johnston JS, Pepper AE, Hall AE, *et al*. 2005. Evolution of genome size in brassicaceae. *Ann Bot (Lond)* **95**(1): 229–235.
31. Budiman MA, Mao L, Wood TC, Wing RA. 2000. A deep-coverage tomato BAC library and prospects toward development of an STC framework for genome sequencing. *Genome Res* **10**(1): 129–136.
32. Cai WW, Reneker J, Chow CW, Vaishnav M, Bradley A. 1998. An anchored framework BAC map of mouse chromosome 11 assembled using multiplex oligonucleotide hybridization. *Genomics* **54**(3): 387–397.
33. Peterson DG, Lapitan NL, Stack SM. 1999. Localization of single- and low-copy sequences on tomato synaptonemal complex spreads using fluorescence *in situ* hybridization (FISH). *Genetics* **152**(1): 427–439.

Copyright © 2005 John Wiley & Sons, Ltd.
